# How the Political Becomes Private: In Vitro Fertilization and the Catholic Church in Poland

**DOI:** 10.1007/s10943-017-0480-3

**Published:** 2017-08-17

**Authors:** Magdalena Radkowska-Walkowicz

**Affiliations:** Institute of Ethnology and Cultural Anthropology, University of Warsaw, ul. Żurawia 4, 00-503 Warsaw, Poland

**Keywords:** Poland, In vitro fertilization, Catholic Church, Private/political

## Abstract

The Polish debate on in vitro fertilization (IVF) is extremely heated and highly politicized. The hierarchs of the Catholic vehemently oppose the use of IVF. In this text, I present the Church’s approach to IVF. Basing on the documentary film, *Three Conversations about Life*, and ethnographic research, as well as an analysis of Vatican documents and official statements of Polish bishops, I show how the positions of clergy might influence private lives. I indicate series of tensions associated with the “politics of morality” of the Catholic Church and the daily lives of people, who have children thanks to IVF.

In 2010, Bishop Tadeusz Pieronek, an important player on the spiritual and political scene in Poland, told a large Internet portal that, “Life born from a test tube is the result of manipulation, and is not the work of nature. Love is not confessed at a store counter. […] The development of nonorganic methods of conception can lead to a future, in which we will order children with specific traits. […] They will be like the creators of Frankenstein. What else is the literary imagining of Frankenstein, a creature called to life in spite of nature, if not the blueprint for in vitro? This is a gruesome perspective, but it exists. […] Couples who decide on in vitro prefer to buy a child rather than adopt one. They don’t want an adoptive child because they desire “their own” child. This is the logic of commodities, not that of gifts (Harpula and Pieronek 2009). At the time, the Bishop’s voice was not unique. For over 2 years already, in vitro fertilization (hereinafter IVF) had been one of the more important political topics in Poland (Radkowska-Walkowicz 2014a), and the public debate over IVF continues without break until this day.

The first, successful IVF in Poland took place in Białystok in 1987. Yet, above all, it was the announcement made toward the end of 2007 that this treatment would be covered from the state budget, which ignited a hot discussion and rendered IVF a matter of politics—to a much greater extent than in other countries. Significantly, until 2015 there was no detailed law regulating IVF treatment. In effect, IVF was primarily regulated by the needs of the free market, as well as by the competences and traditions of Polish reproductive medicine. Once the law on infertility treatment was enacted, however, the discussion did not die out. Instead, it has grown to focus less on the conditions and limitations that should govern the use of this fertilization method, and more on whether IVF should be allowed in Poland, at all.

The main actors in this discussion are: the hierarchs of the Catholic Church, which calls upon Vatican documents to vehemently oppose the use of assisted reproductive technology (hereinafter ART) methods, conservative party politicians and their allied publicists, doctors as well as NGO activists. Those who have used IVF and the children born using this method rarely participate in the discussion.

IVF critics employ very strong rhetorical measures, in order to point to the “lack of dignity” and “inhumanity” that typifies the use of ART, as well as its “unethical” character. They talk about murder, holocaust, and claim that children born thanks to IVF suffer from physical, social, and mental health problems (por. Radkowska-Walkowicz 2012). The only visible narrative that responds to these accusations uses heavily medicalized language, and portrays ART (assisted reproductive technology) methods as neutral and not subject to discussion from any perspective other than the medical. In effect, a process of neutralization takes root, in which infertility and in vitro technology are regarded as only belonging to the field of medicine, and as not associated with issues of ethics or culture (such as blood relations, reproductive tourism, or biomedicalization).

The conflict over IVF must be seen through a wider, political, and historical lens. Of particular importance here is the systemic transition of 1989, Poland’s entry into the European Union, and the growing influence of the Catholic Church in Polish politics during the last years. The history of the conflict over IVF sheds light on the various tensions in the process of the Polish transition; it is a microhistory, one part of the “grand” history of Poland after 1989. In such context, it becomes clear that this conflict engages not only the medical, moral, and legal spheres, but also the concept of citizenship, the nation, the state, and the role of the Catholic Church within (Radkowska-Walkowicz 2014a; Holc 2004; Mishtal 2015). As in the case of the discussion on abortion, the discussion on IVF “represents a coded discourse that reflects fundamental concerns, including the shape of the state itself, the state’s obligation to society (and vice versa), the rule of law, and […] the scope of the protection of civil rights and fundamental freedoms (Zielińska 2000: 24).” The conflict over IVF thus represents a terrain, on which political actors negotiate the national “moral code” (Mayer 1999: 3), the gender norms, as well as the conditions of participation in the “imagined community” (Anderson 1983; see Radkowska-Walkowicz 2014a). Specifically in the Polish context, this conflict is about the Catholic Church’s sphere of influence and the weight of its moral guidelines on politics, the law, and the lives of ordinary people; at stake is not so much ethics or medicine, but the Church’s position in the political game of power and its right to moral governmentality (Mishtal 2015).

In this text, I present the Church’s—especially the Polish Church’s—approach to IVF. Departing from the documentary film, *Three Conversations about Life*, I show how the positions of Catholic clergy might influence private lives. This film very accurately portrays the complicated relation between the private and the political in the Polish “in vitro experience.” In order to show how the private sphere is encroached upon, I call on the accounts of people who have children thanks to IVF.

This text is based on anthropological research on IVF and utilizes the multi-sited ethnographic approach proposed by Marcus (1995) which examines the topic from diverse angles, including discourses, experiences, and perspectives on IVF.^1^ I have conducted it over the past eight years. In my prior work, I focused mainly on women’s experiences of infertility, on the various aspects of the Polish debate on IVF, as well as on the category of the “in vitro child,” its political status, and the identity of concrete individuals (bibliographical data—anonymization). The research, which I conduct along with my team, has thus far concerned more than 100 people, including infertile women and men, children born thanks to IVF as well as their siblings, grandparents, and over 20 doctors who specialize in reproductive medicine. With the majority of these interlocutors, we conducted in-depth interviews. Further, we conducted focus group interviews with children, a series of participatory observations, and media discourse analysis. In this text, I base my discussion primarily on the Catholic Church’s official documents. I also analyze statements made by persons affiliated with the Church, including hierarchs and Catholic publicists. A second source for this text is the documentary film I have already mentioned and a public debate with its director that took place in Warsaw on November 24, 2016. I also employ interviews and analysis of Internet forums, conducted within the frame of my research projects.

## The Catholic Church and IVF

To a significant extent, the conflict over IVF constitutes a conflict over the position and the role of the Catholic Church in Poland. More specifically, it concerns maintaining the Church’s “monopoly on morality” (Inglis 1998), which is strictly associated with control over the family, sexuality, and fertility. This, in turn, can translate to control over the everyday moral choices of Polish people (even, as I indicated, the influence of Church on moral choices of Polish people is not straightforward and widespread), and over the state and its institutions, especially those involved with medical interventions (Mishtal 2015). Church hierarchs, regular priests, many activists, and Catholic publicists take a stand on IVF. The main source of legitimization for the perspective that they represent are the official teachings of the Church. Another source is scientific studies, summoned in a manner characteristic of popular publications (Dolińska 2009). Defense of the human embryo, also in the context of IVF, is a matter discussed in many of the Catholic Church’s documents, among them in the documents of the Congregation for the Doctrine of the Faith, in particular in the instructions of the *Donum vitae* (*Instruction on respect for human life in its origin and on the dignity of procreation, replies to certain questions of the day*), the more recent *Dignitas personae: On Certain Bioethical Questions*, the encyclical *Evangelium vitae*, and in the *Catechism of the Catholic Church* (see Szymański 2009). A close reading of these documents allows for understanding the immense influence that they have on the formulation of judgments concerning IVF, in the rhetoric of its opponents.

And so in the *Donum vitae* we read:

Thus the fruit of human generation, from the first moment of its existence, that is to say from the moment the zygote has formed, demands the unconditional respect that is morally due to the human being in his bodily and spiritual totality. The human being is to be respected and treated as a person from the moment of conception; and therefore from that same moment his rights as a person must be recognized, among which in the first place is the inviolable right of every innocent human being to life. […] since the embryo must be treated as a person, it must also be defended in its integrity, tended and cared for, to the extent possible, in the same way as any other human being as far as medical assistance is concerned. (Ratzinger 1987)

The Church also takes a stance on the matter of freezing embryos:

*The freezing of embryos,* even when carried out in order to preserve the life of an embryo - cryopreservation - *constitutes an offence against the respect due to human beings* by exposing them to grave risks of death or harm to their physical integrity and depriving them, at least temporarily, of maternal shelter and gestation, thus placing them in a situation in which further offences and manipulation are possible. (Ratzinger 1987)

Further, the Church preaches that human life should be passed on only in marriage (“The child has the right to be conceived, carried in the womb, brought into the world and brought up within marriage”, which clearly makes heterologous fertilization—assisted fertilization of woman’s ova with donor sperm—unacceptable). What’s more, “even in a situation in which every precaution were taken to avoid the death of human embryos, homologous IVF and ET [embryo transfer] dissociates from the conjugal act the actions which are directed to human fertilization” (Ratzinger 1987), also making it incongruent to Church teachings.

Homologous IVF and ET are brought about outside the bodies of the couple through actions of third parties whose competence and technical activity determine the success of the procedure. Such fertilization entrusts the life and identity of the embryo into the power of doctors and biologists and establishes the domination of technology over the origin and destiny of the human person. Such a relationship of domination is in itself contrary to the dignity and equality that must be common to parents and children. (Ratzinger 1987)

Masturbation also turns out to be a problem, associated with in vitro technology: “Masturbation, through which the sperm is normally obtained, represents another sign of this dissociation: even when it is done for the purpose of procreation, the act remains deprived of its punitive meaning” (Ratzinger 1987). Of note here is that in accordance with The Pontifical Council for the Pastoral Care of Health Care Workers, artificial insemination using the husband’s seed is acceptable, under the condition that the sperm necessary for fertilization is obtained from the sexual act of a married couple. Obtaining sperm, however, requires a condom, which in other circumstances would be considered an illicit means of contraception. In this case, as in cases when sperm must be the subject of a medical examination, the use of a condom is allowed, so long as it is punctured. “A perforated hood, which unlike the traditional condom does not contain spermicides and allows sperm to penetrate the woman’s vagina, gives the feeling of openness to fertility,” writes father Jerzy Szyran (2009: 47). If sperm is obtained from sex, the LTOT (*low tubal oocyte transfer*) method is permitted, which involves the transfer of the female gamete from the ovary to the Fallopian tube or the uterus, where it can be inseminated. Another approved method is the GIFT (*gamete intra*-*fallopian transfer*) or the simultaneous transfer of both gametes to the Fallopian tubes (Sporniak 2006). Nonetheless, the fact that the Church allows such technologies is entirely omitted in media accounts authored by Church hierarchs or allied publicists.

Representative of the approach of the Polish Bishops as well as of many Catholic publicists seems to be the statement published by the Team of Experts on Bioethics of Polish Episcopal Conference in concurrence with the Presidium of Polish Episcopal Conference. Here we read,

Human dignity is encroached upon in nonorganic fertilization procedures since conception occurs not during the act of love, but in the course of experimental technical procedures. This carries the signs of “human production”. […] we justify opposition to IVF also basing upon natural law, or the data of universal knowledge, commonly recognized norms, to which all people, regardless of perspective, are obligated. […] The killing, selection and freezing of human embryos is morally unacceptable. They are human beings, who deserve full legal protection, especially the protection of the right to life (Oświadczenie Zespołu 2010).

Although the Vatican’s critical stance on IVF is clear, church hierarchs in Poland appear to have zeroed in on reproductive issues, going far beyond their colleagues in other European countries. My research shows that the politicization of the IVF issue in Poland is unique and that the Polish episcopate is very conservative (Hall 2016: 60). Because of the nature of Polish democratization after 1989 in which the Church took full advantage and gained real political power (Nowicka 2007), and the fact that Catholicism is dominant in Polish society—about 92% of children are baptized according to Catholic tradition (Mariański 2010)—the Church wields significant influence on public debates, as well as on the legal and other initiatives of Polish politicians. In the past year, the Church’s sphere of influence has been further enlarged (meaning in 2016, due to the election of the populist–nationalist PiS party, which is strongly allied with the Church). Simultaneously, paradoxically, nonorganic fertilization methods are widely supported by Polish society: according to studies conducted since the 1990s by the Center for Public Opinion Research (hereinafter CBOS), more than 75% of Poles accept the use of ARTs by married couples and the majority also accepts their use by unmarried couples and single women (CBOS 2015). These data point to the strongly pro-family character of Polish society, where the family represents the most important point of reference, while offspring are considered fundamental to a happy life and the fulfillment of one’s potential (Radkowska-Walkowicz 2013: 197–201).

The Church’s conservative stance is not fully adhered to by Polish society because, in following the wider tendency in Europe, Polish society has undergone a general moral liberalization. And so, Poles harbor open attitudes toward matters of sex (such as premarital sex, non-monogamous relationships, or contraception), among others. What’s more, a substantial individualization and privatization of religion have taken place in Poland (Luckmann 1967; Hall 2012). “I conduct a conversation with God, but maybe these are conversations with myself, I don’t know,” told me one of my interlocutors, which serves to illustrate this tendency. Although to proclaim an institutional crisis in the Catholic Church would not be factual, many church goers reinterpret church dogmas and teachings according to their own point of view. As writes Irena Borowik, the most characteristic feature of religiosity in Poland is the one-of-a-kind combination of, “a very strong feeling of duty (orthodoxy) in the field of rituals, a general societal identification with Catholicism […], proximity of the Church to the individualization and privatization of the sphere of religious beliefs, and to that of ethical substantiation (Borowik 2001, s. 152).”

A person’s relation to faith and the teachings of the Catholic Church, according to a CBOS report, translates to the level of acceptance of IVF:

Almost every other Pole (46%) declares themself as a person of faith, who follows Church doctrines. A nearly identical in size group (47%) is comprised of people who believe, but in their own manner. Only a few consider themselves to be agnostics: they are, however, unable to clearly define themselves as believers (3%) or nonbelievers (altogether 4%). The approach to in vitro fertilization appears in the least definitive among respondents belonging to the first of the mentioned categories […]. Yet this group also clearly exhibits support for using this technology by married couples struggling with the problem of infertility […] acceptance for the use of this method by people who declare to follow Church doctrines does not derive from their ignorance of the Church’s stance on this matter (CBOS 2010, s. 3).

Whereas many Catholics undergo IVF treatment, a considerable amount of my interlocutors left the Church due to its stance on IVF. Words of my interlocutor describe well the relation that many young Poles have with the Church.

On paper we are Catholics and we are raised in the Catholic tradition, we’ve been living in this tradition. […] Our child is baptized, although here is where my husband and I disagree, because he is a little bit more upset with the church than I am. Meaning, frankly, I just don’t care. Let them say whatever they want to […] it doesn’t affect me anymore, of course it makes me angry, but it doesn’t hurt me.

The voices of other, non-Catholic, Churches are completely silenced in Poland; the media take no interest in them, and neither do lawmakers. Although the distribution of power and knowledge toward and among the faithful differs from church to church, on the whole it appears that other religions present in Poland have adopted a more open approach to reproductive technologies (Inhorn and Tremayne 2016; Kahn 2000; Radkowska-Walkowicz 2013: 61–62).

The presidential election of 2015 testifies to the strong affinity between the political sphere in Poland and the Catholic Church, along with its stance on IVF. The presiding president Bronisław Komorowski dedicated one of his campaign ads entirely to the issue of IVF. In it, he argued that his opponent (who later won the election) stood for the complete penalization of IVF, including prison sentences for its users and doctors. When the president Komorowski signed the bill regulating IVF treatment, some voices called for his excommunication. In response, The Legal Council of Polish Episcopal Conference released the following statement:

The IVF bill, adopted by the Polish Parliament- for the price of a child’s birth- infringes both upon the lives of other children conceived during this procedure, and upon the inviolability of human life from conception to natural death […]. For this reason, the bill cannot, in any way, be accepted by a Catholic. The president of the Polish Republic and the members of parliament who supported the IVF bill, in so doing publicly expressed their opinions, which are at the source of serious wrongdoing to many of the faithful. This represents “an attitude or behavior that leads another person to commit evil (KKK, 2284)”. A Catholic who consciously and willingly signs or votes for the acceptance of IVF procedures undermines the communion, or his own full community with the Catholic Church (Dzięga and Maćkowski 2015).

In the statement’s third point, the authors asserted the following:

As for persons acceding to the holy sacraments, according the Codex od Canonical Law, “He who is conscious of heavy sin, should not without confession […] receive the Holy Communion” […]. Thus, if someone consciously and willingly stood against human dignity by voting or signing the in vitro bill, and if this person should want to receive the Holy Communion, first he should reconcile with God and the community of the Church through the sacrament of reconciliation, express his repentance due to the committed sin, assert that he will improve and finally, rectify his deed. […] such a person should refrain from receiving the Holy Communion until he publicly changes his position (Dzięga and Maćkowski 2015)

At the same time, the authors recognized that voting for or signing the IVF bill could lead to automatic excommunication, because only an official could rule excommunication after studying the matter in detail (Dzięga and Maćkowski 2015).

## The Public Becomes Private

The IVF debate not only plays out in the field of politics, but also takes place in private homes. The private is political, as feminism has taught us; meanwhile, the political also intervenes in the private order of things. And although according to CBOS data the percentage of respondents who support IVF remains very large, tensions surrounding the technology are likewise mounting in Polish families. My research among persons who experienced infertility and were treated for their condition (Radkowska-Walkowicz 2013) suggests that in many cases, the issue is kept hidden as a family taboo. Often time, this is not only related to the stigma of infertility (which still exists and is not specific to Poland), but also to the politicized realm of IVF procedure. In practice, for Catholic families this translates to the necessity of dealing with the Church’s disapproval, in addition to the fear of being stigmatized as parents and children.

Various scenarios of family tensions and conflict associated with IVF and the attachment to Catholic religion are possible. Some of my interlocutors did not tell their parents that their grandchild was born thanks to IVF. Others enjoyed full or partial support from their loved ones (including financial support), while in certain homes, although the parents and grandparents know about it, the matter is not talked about [for more on silence in the context of the IVF experience see: Allison (2011), Radkowska-Walkowicz (2013)]. In the book, “In vitro: Without fear, without ideology,” Anna Krawczak, the former president of the Association for the Treatment of Infertility and Adoption “Nasz Bocian” (“Our Stork”) and a member of the European Society of Human Reproduction and Embryology, describes her father’s approach to the issue:

A deeply devout man; it was he who had sent me to those oasis camps. He has many friends among the priests, and he starts and ends his day with prayer. Having discerned our pains, about which we were not profusely vocal, he handed the necessary sum for in vitro treatment, saying: “So that I can become a grandfather for a second time” (Krawczak 2016: 34).

Wanting grandchildren and the attachment to a traditional family formula ultimately lead to working out such a stance vis-à-vis the Church, which allows for accepting the choice of reproductive medicine without leaving the Church. Karolina Wolf, a teenager born thanks to IVF, recounts her story to the press, “When my mother took me to church because she wanted to baptize me and she just told the priest about it, he said: ‘But you know, that’s not possible, this is a child without a soul, it has no right to exist’ (Wolf 2012).” Officially, the Catholic Church does not condemn IVF children. In his July 2015 sermon at Jasna Góra Monastery (it is the home of the Black Madonna painting—an image which has often been seen as symbolizing the Mother of the Nation—and is a very famous pilgrimage destination, also for politicians), Archbishop Andrzej Dzięga explained the matter as follows: “It is not your fault that for one of you to be born, a couple dozen other children had to die or be killed. This is not your fault—the adults will answer to God for this, those who ordered this, those who proposed this.”^2^ On the Internet portal, “Gazeta.pl,” a female user, writes:

I went to see the parish priest yesterday. I learned that I live in sin and that I will wallow in it for the rest of my life. That my child and I will be punished for what I have done, that the fact that my child is healthy now does not mean that it won’t become sick after a while and that I shouldn’t be surprised then because this will be the punishment, which will catch up with me either way.^3^

My interlocutors also told me about more lenient priests, who asserted that the means of conception is of no interest to them. Yet the majority did not admit in church to having used IVF, considering it neither relevant, nor a sin. But the Church’s preaching cannot be entirely ignored: information about it appears in the media, often time during religion classes at school, and sometimes even random people mention the “evils of in vitro.” For my interlocutors, especially those who are children, the Church’s stance is received as stigmatizing and totally unjust.

Agnieszka Ziółkowska, the first Pole born thanks to IVF (in Italy in 1987), announced that she will apostatize due to the Church’s position on IVF (Ziółkowska 2013). Her decision was widely commented upon by both rightwing and liberal media. Agnieszka’s mother also decided on apostasy, despite declaring that her child was born “due to prayer.” Maria Bąk-Ziółkowska writes, “They implanted three embryos. When they did this, the bells tolled for the Angelus. And me… (wipes her tears). I had this papal rosary. I clutched it and prayed that it would go alright (Bąk-Ziółkowska et al. 2015).” Agnieszka’s father admits that after hearing Archbishop Dziega’s sermon, he did not go to church on Sunday for the first time. The political expression of Church rules in various ways impacts on private life of Poles. For some, it can mean necessity of weakening the role of Church in their life, for other—weakening the family ties.

## Three Conversations About Life

One of the more interesting voices concerning the relationship between the Catholic Church and IVF in the context of its influence on family relations and the practices of daily life is the documentary *Three Conversations about Life*. This short film was produced in 2016 by Julia Staniszewska, a visual artist, in her directorial debut. The producers (The Munk Studio—SFP and Kijora) present the documentary as follows:

A difficult and painful conversation between a mother and her daughter. The mother- a doctor, a devout and practicing Catholic. The daughter- an agnostic, her children were born thanks to in vitro. Her mother loves her grandchildren, but does not accept this technology. Her daughter’s dream is that her mother will change her mind. The mother is torn between her love for her daughter and grandchildren, and Church dogma. She wants her daughter to try and understand her. The film tries to understand them both.

For me, the film is first and foremost a story about a family, which came about thanks to IVF, and about relations, between a daughter and a mother, who is a practicing Catholic, and between a loving grandmother and her grandkids. This is a film about treating infertility and attempts to understand a mother, who does not accept IVF. It is also a film about a world, which became more complicated with new reproductive technologies and old hierarchies of value. The film is composed of three parts, each made in a different year and each showing a meeting between the mother and daughter. The first meeting presents a downright fight. The daughter accuses her mother of not accepting IVF, thanks to which her grandchildren were born. The mother defends herself: she cannot accept it, because the Church, here synonymous with God, does not accept it. In this part, we hear one of the film’s more important dialogues: “Would you have preferred it if I had adopted a child?” asks the daughter, to which the Mother answers: “As a religious person, yes.” With these words, the woman not only confirms her strong disapproval of IVF, but also, in distancing herself with the formulation “as a religious person,” she locates a certain conflict within herself and shows how torn she is inside. Can she accept it then, as a mother and a grandmother? The answer appears to be “no.” It seems that her identity (as a religious person) and her location in the framework of the Catholic value system do not allow her to fully and unconditionally accept an IVF child. During a discussion about the film, Julia Staniszewska commented her mother’s stance as follows:

That was a breakthrough moment, […] to stop fighting for myself as a daughter, and to start thinking that I have in front of me my mother, who is religious and has this hierarchy of values. And later on she says in the film: “If God comes in the first place then everything else falls into its place”. And the first image that came to my mind was Abraham. And the child as sacrifice. (Staniszewska et al. 2016)

This would agree with the understanding of the tale about Abraham and Isaac proposed by Soren Kierkegaard in “Fear and Trembling.” In a certain sense the mother, like Abraham, is “an emigrant from the sphere of the universal” (Kierkegaard 1985: 139), thus from the sphere of ethics and rules, which govern our moral institutions. That the mother will not question the Church becomes obvious once she says, “One can’t describe themselves as a person of faith and at the same time say that some doctrine is good and acceptable and reject some others because they don’t suit them personally. You need to be consistent.” And later, “I have a damn difficult role here—what’s more important? Your child or God and the church?” Julia asks her mother, “your love for God is more important than your love for your child?” The mother answers, “Darling, you know I love you very much, but I can disapprove of your behavior, right?” When her daughter asks her what she will tell her grandkids, when they ask her: “Why doesn’t grandma approve of IVF?” The grandmother says, “Because… other children die.” In the film’s second part, the daughter tells her mother, “Instead of turning directly to me, right here, you turned to God.”

The mother’s determination is not typical of members of the Catholic Church in Poland. Although, as I have already mentioned, while declared Catholics constitute a substantial majority in Poland, similarly to many other countries the Polish model of Catholicism is based in large on a selective approach to the dogmas of the Church. The privatization of religion is also a fact. Further, in response to the Church’s restrictive policies concerning reproduction, women “develop their own *unofficial biopolitics*.” (Mishtal 2015: 159)

The film does not directly explain the mother’s radicalism and her unconditional obedience to Church dogmas (but the director confirmed my intuition about this). The mother is a member of the Neocatechumenal Way, a Catholic community whose statutory aim is to promote the renewal of parish life in the spirit of the Second Vatican Council. As writes Hanna Frejlak (2016), who conducts research among Polish members of the community, in accordance with its teachings, all relationships other than that with God are pushed aside. Characteristic of this group is the deep internalization of the first commandment (*Thou shalt have no other gods before me*) understood to indicate not only literally, other gods, but also things, money, goods as well as other people. Importantly, the Neocatechumenal Way also denotes a certain life philosophy, which organizes family, social, and political life. Ingrained here is a radical approach to sex and reproduction, in accordance with the teachings of the *Humane Vitae,* the encyclical written by Pope Paul VI. Julia’s mother is thus not a “typical” Polish Catholic, but a member of a community, which does not allow for religious privatization or leave room for negotiation when it comes to Church dogmas and guidelines. Being a part of this community is totalizing and at the same time congruent with the Catholic order of things.

In spite of her involvement in the religious structures of the Neocatechumenal Way, the mother is deeply torn, to which attest the last scenes of the film. “It affects me at such a deep level, darling,” she says to her daughter. Although everything “ends well” and family life continues, perhaps like in Abraham’s case in following Kierkegaard’s interpretation, the mother’s “eyes were darkened,” and she “saw joy no more.” It is the mother who is a truly tragic heroine and it appears that Julia comes to understand this at the end of the film. During the discussion about the film, she noted

And this is not just my problem, because me, well, my husband and I were successful, we have children. I don’t have a problem with IVF, neither in the moral sense, nor ethically. And I see before me my mother, whom I love, who has a real, very deep dilemma. And at this moment, suddenly from this anger that was inside me, with which I began this film, and the bitterness with which I come to her, and then I felt that really, I feel bad for her. (Staniszewska et al. 2016)

The relation is reversed. Julia could be seen as a victim of biomedicine and of secularized modernity—that is surely how the Church would surely like to see her, often basing its claims on just this kind of argumentation. “Can a woman really be happy, when she is treated like a birthing cow,” asked Urszula Dudziak (2008), a Church-affiliated professor at the Catholic University of Lublin. Meanwhile, Wanda Półtawska rules: IVF “reduces humans to the role of animals […] a woman is not a breeding animal.”^4^ Julia, as she convinces, does not have a problem with IVF, she is a happy mother, an artist, and she does not feel like a victim. In a certain sense the victim is her mother, who like Abraham, only has silence left: “Abraham remains silent—but he *cannot* speak. Therein lies the distress and anxiety” (Kierkegaard 1985: 113).

The story about Abraham can of course be read completely differently (on the quantity of interpretations see Kalimi 2010), and the mother can be seen as the enslaved, blind follower of an external authority. Simultaneously, leaving biblical history aside, the film might be interpreted as a story of the mother’s emancipation from unconditional devotion to her daughter, from totalizing motherhood. Thus, the mother must choose between enslavement on the one hand and liberation on the other. Julia Staniszewska admits that for her, the film represents a process of growing pains, from “being a daughter, to being a sister or a partner.” The daughter’s growing up translates to allowing her mother freedom.

## Medicine

In the second part of the film, we learn that the mother is a doctor. She believes in medicine, but she is also aware of its limitations. The daughter remembers one of her own contacts with medicine: her body was fragmented into pieces, and she felt that she does not understand doctors and that they do not want patients to understand. She needed then a mother–doctor, just like the time when she had a miscarriage. The figure of the doctor shows well that science does not lie at the center of the conflict over IVF. A simple binary of desacralized biomedicine on the one hand and irrational/unscientific religious ideology on the other does not exist here. IVF opponents often call upon science, basing their convictions on arguments surrounding women’s and children’s health. Yet as Barbara Dolińska has argued in the magazine “Science” (2009: 96–97), their line of reasoning is most often seriously flawed as they cite outdated research or fail to provide sources for their data (for more on this see: Radkowska-Walkowicz 2012). Science is not in competition with religious language here; on the contrary, it serves for legitimation. Perhaps, the best example is “naprotechnology”: promoted and approved of by the Catholic Church, this method uses evidence based medicine, alternative medicine, complementary medicine as well as very detailed observations of the female body, such as of the vaginal discharge (for more on this method see: Radkowska-Walkowicz 2014b). The Catholic daily *Nasz Dziennik* printed Archbishop Ignacy Dec’s commentary after the announcement of the Nobel Prize winners in the field of medicine, Robert G. Edwards and Patrick Steptoe, who in 1978 led to the first successful birth of a child conceived through IVF:

Again, science has been enslaved. It does not serve the truth, but rather, it is at the mercy of ideology […]. Every instance of the enslavement of science through history has turned out to be very harmful. […] We had examples of this in the totalitarian systems. In the postwar period, science was again enslaved, but this time by economics. […] It is a great pity that modern people of science so often bend to the pressure of politicians, journalists, or, in short, allow themselves to be ideologized. And that means departing from the truth (Dec and Jagodziński 2010).

Science so defined is not simply that which scientists bring. Scientists are no longer the depositors of knowledge, especially if their opinions run on the contrary to the idea that the meeting of two gametes denotes the beginning of human life. This sort of science would be fully in accord with the morality prescribed by the Catholic Church. In this rhetoric, the world has not been disenchanted for good since science has turned out to be elastic enough so as to legitimize religious dogmas and papal interpretations. This romance between the Church and science is nothing surprising. Church documents provide for this relationship, like John Paul II’s encyclical letter *Fides et ratio*, which begins with the words: “Faith and reason are like two wings on which the human spirit rises to the contemplation of truth” (John Paul II 1998). In observing the Polish debate on IVF, we notice not only the civilizational and disenchanting character of Catholicism that derives from its Judeo-Christian roots (Habermas 2005, Bielik-Robson 2008: 63–73), but to an equal extent its political conditioning. Science is at once employed in a manner approved of by the Church’s modern guidelines, and often time serves as a reinforcement on the political battleground.

## Conclusion

As I have discussed in this text, family histories of IVF are diverse and the attitude toward this medical technology can be the cause of many tensions. Generally, I can point to three models of relations among Catholic family members in the context of IVF: (1) Silence: conservative parents are not informed about the fertility issues of their child and even though they know about his/her IVF experience; this subject is not raised in the family. (2) Conflict: the Catholic member of the family does not accept IVF and openly expresses his/her perspective. Such is the relation between Ewa and her brother, who “is a very radical Catholic.” She told me about their argument, recalling that “many things were mentioned, that it’s my own fault because I waited too long for my second pregnancy. (…) He can’t relate to my situation. Of course he talks about frozen embryos and etc., about murdered babies too.” (3) Acceptance and support. Many of my interlocutors, including the grandfathers and grandmothers of “IVF children,” told me that they do not understand the position of “their” Church: “What does that church want from in vitro? In vitro is a real source of joy”—said Tomasz. In the documentary, which I interpret in this text, the situation between the first and the second models is shown. The silence is broken by the camera, but its penetration and Julia’s tenacity cannot change the mother’s attitude toward the Church and reproductive technologies.

In the broader context, after the systemic transition of 1989, the Polish Catholic Church began to greatly interfere in state politics and in the everyday choices of Polish people. This “multi-level infiltration of Catholic ideology into public and private spaces” (Mishtal 2015: 66) principally concerns morality, as well as the management of reproduction. In the past years, the IVF debate has become “the new abortion debate” (Chełstowska 2011: 104) and another field for disciplining the citizenry. Attempts to render women’s bodies docile occur simultaneously in public—by enforcing laws, regulations and managing the distribution of funding—and private space. The Church reaches the latter through the media, but also through direct meetings held in private homes (for example during the *kolęda* ritual, or priests’ pastoral visits to people’s homes), at church, during confession, baptisms, Catholic community meetings, or mandatory premarital courses (Mishtal 2015). The infertile persons who decide to undergo IVF are very often Catholic. The Polish model of religiosity allows them to reconcile the Church’s dogmatic rejection of IVF with the desire to treat their infertility. But there remain other actors, who are also affected: the children, who feel stigmatized and excluded by the Church, or the grandparents, who are often more strongly aligned with the Church but who are simultaneously extremely attached to their grandchildren, with whom they usually maintain very close relations. My research points to a series of tensions associated with the “politics of morality” (Mishtal 2015) of the Catholic Church and the daily lives of people, who have children thanks to IVF. Identifying these tensions can help better understand the specificity of the Polish family, as well as the mechanisms of the penetration of religious rules into the private sphere. Finally, it helps to shed light on the complicated relation between the Catholic Church and the state in Poland.

## Footnotes

1 The multi-sited ethnographic approach suggests the following relations and associations (Marcus 1995, 97) as a response to the fragmented reality of modernity, as well as to examine the dispersed, ambiguous, and multi-sourced identities of contemporary people. A multi-sited ethnography thus makes it possible to ‘capture distinctive identity formations in all their migrations and dispersions’ (Marcus 1998: 63).

2
http://wpolityce.pl/kosciol/259018-wazne-slowa-abp-dziegi-na-jasnej-gorze-ustawa-o-in-vitro-jest-zbrodnicza-bo-odmawia-sie-w-niej-czlowieczenstwa-zyjacemu-i-poczetemu-czlowiekowi-to-jest-czysta-eugenika.

3
http://forum.gazeta.pl/forum/w,191,131940396,,zaraz_ide_do_proboszcza_i_ich_rozniose_chyba_.html?v=2; dostęp: 20.01.2013.

4 Statement made during a conference organized at the Cardinal Stefan Wyszyński University in Warsaw: http://www.kosciol.pl/article.php?story=2009032116534754.
